# Complete mitochondrial genome sequence of historical olive (*Olea europaea* Linnaeus 1753 subsp. *europaea*) cultivar Mehras in Jordan

**DOI:** 10.1080/23802359.2023.2275828

**Published:** 2023-11-07

**Authors:** Monther Sadder, Mohammad Brake, Salam Ayoub, Yahya Abusini, Ibrahim Al-Amad, Nizar Haddad

**Affiliations:** a School of Agriculture, The University of Jordan, Amman, Jordan; b Science Faculty, Jerash University, Jerash, Jordan; c National Agricultural Research Center – NARC, Baqa’a, Jordan

**Keywords:** Mehras, mitogenome, olive

## Abstract

The complete mitochondrial genome of the olive cultivar Mehras was determined using high-throughput sequencing technology. It consisted of 710,808 base pairs and comprised 70 genes, including 44 protein-coding genes, 23 tRNA genes, and three rRNA genes, with a GC content of 44.7%. Significant single nucleotide polymorphisms (SNPs) and insertions/deletions (InDels) were detected throughout the mitogenome. Phylogenetic analysis was conducted using other genotypes, including five olive cultivars, three related species, and *Olea exasperata* as an out-group. The analysis revealed that the olive cultivar Mehras shares an ancient common ancestor with the Frantoio cultivar from Italy and the Manzanilla cultivar from Spain, which confirms previous findings based on plastome sequencing.

## 1. Introduction

Olives are increasingly gaining importance worldwide, expanding beyond their original roots in the Mediterranean basin (Besnard et al. 2011; Haddad et al. 2021). The historical olive cultivar Mehras from Jordan (Haddad et al. 2021) is garnering increased attention as a national geographical indicator and a brand associated with 1000-year-old living olives in Jordan. While several olive plastomes have been published (Besnard et al. 2011; Haddad et al. 2021), the availability of mitogenomes is limited. Therefore, our objective is to assemble the Mehras mitogenome as part of the Mehras genome project, aiming to explore its potential for involvement in breeding programs.

## 2. Materials and methods

**Figure 1.**
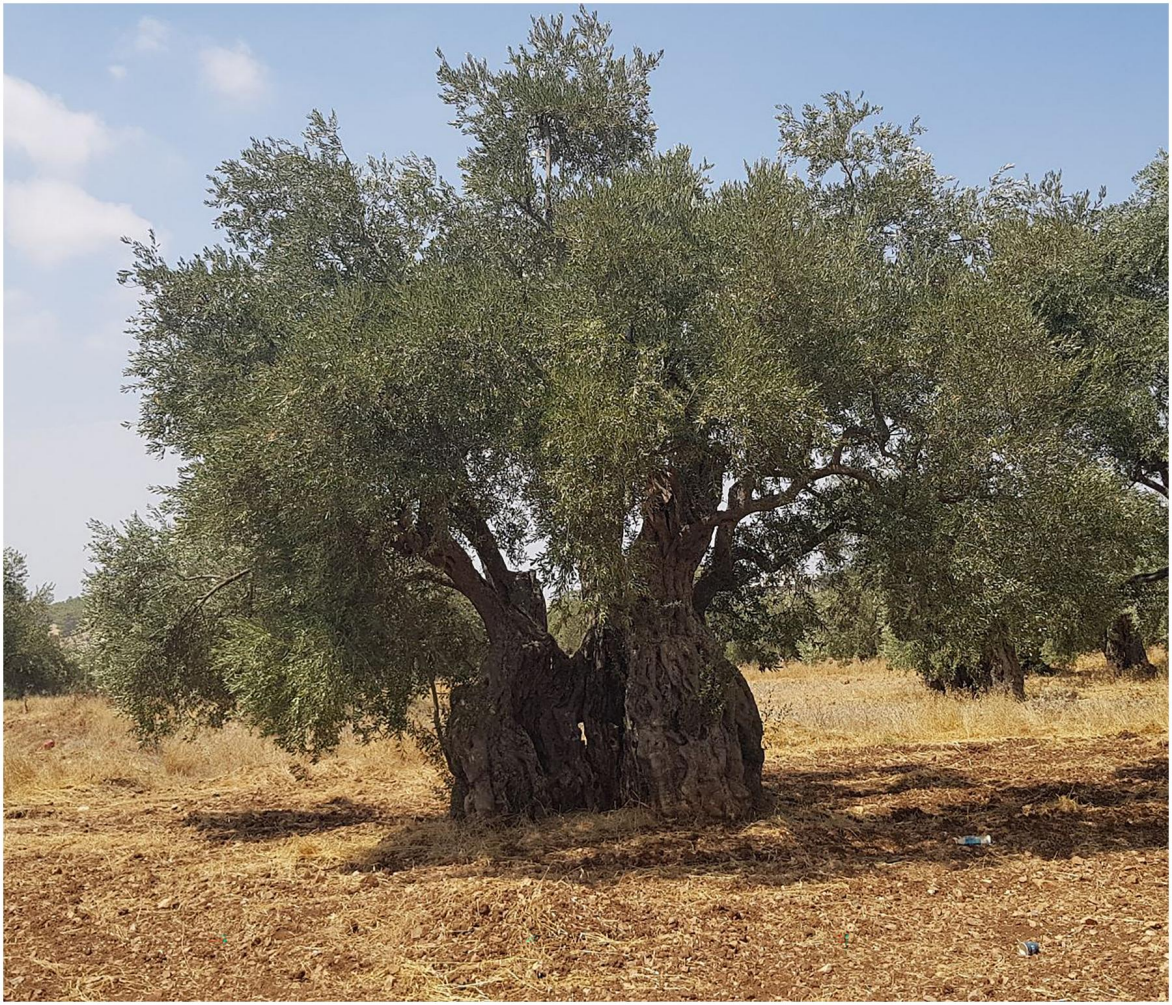
A reference image of the historic olive cultivar Mehras. Photo courtesy of Dr. Salam Ayoub.

Leaf samples from the olive cultivar Mehras (Figure 1) were collected in Alhashemya, Ajloun, Jordan (32.365906N, 35.663445E). A specimen was deposited at the University of Jordan (Monther Sadder: sadderm@ju.edu.jo) with the voucher number JU2023_05. Genomic DNA was isolated using a kit from Promega (Madison, WI) and subsequently sequenced using 64 bp paired-end reads (Illumina, San Diego, CA). The assembly of Mehras mitogenome was started by trimming Illumina reads. Thereafter, they were mapped to the closest olive mitogenome available in the GenBank, which was Stavrovouni Monastery 11, accession MG372117 (NCBI 2023) published by Besnard et al. (2011). The mapped reads were used to build one contig followed by sequence annotation. All assembly steps were performed using CLC Genomics Workbench (Redwood City, CA). The sequencing depth averaged 1648x (Supplementary Figure 1), and all complex genes were successfully resolved (Supplementary Figure 2). The Mehras mitogenome was visualized using PMGmap (http://47.96.249.172:16086/home/) and OGDRAW (Greiner et al. 2019).

Phylogenetic analysis was conducted using 52 protein-coding genes and introns, which are listed in Table S6 of Van de Paer et al. (2018). Five olive cultivars (*Olea europaea* Linnaeus 1753 subsp. *europaea*) were included in the analysis, consisting of three previously published mitogenomes (MG372117 from Cyprus, MG372118 from France, and MG372119 from Portugal) and two mitogenomes assembled in this study using SRA data (SRX6614303 from Italy and SRX6614320 from Spain). Additionally, three related species were included in the analysis (MG372116 – *O. e.* L. subsp. *cuspidata*, MG372120 – *O. e.* L. subsp. *guanchica*, and MG372121 – *O. e.* L. subsp. *laperrinei*). As an out-group species, *O. exasperata* was utilized. The sequences were aligned using CLC Genomics Workbench (Redwood City, CA), bootstrapped 1000 times, and employed to generate a maximum-likelihood phylogenetic tree using PHYLIP (Felsenstein 2004).

## 3. Results and discussion

**Figure 2.**
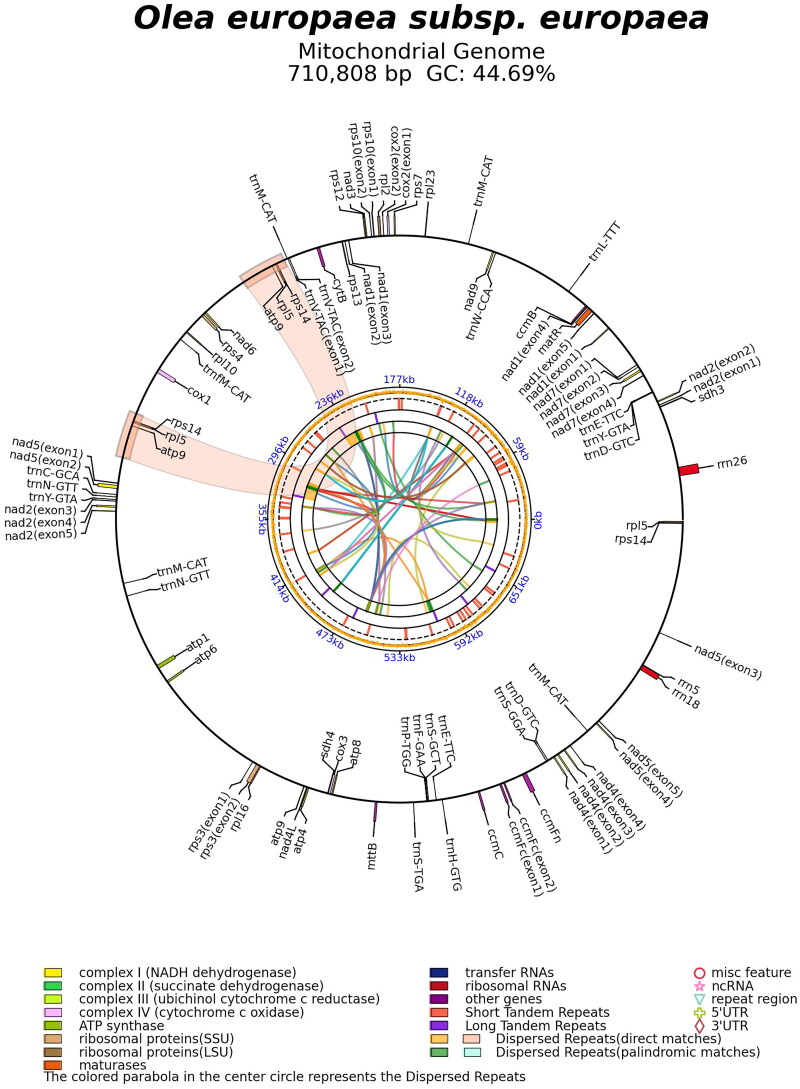
The complete mitogenome of Mehras cultivar. Key showing gene families.

The mitogenome of Mehras is a circular molecule with a length of 710,808 bp (Figure 2), containing the typical genes found in plant mitogenomes. It comprised of a total of 70 genes, including 44 protein-coding genes, 23 tRNA genes, and three rRNA genes, with a GC content of 44.7%. Interestingly, similar to *Olea* species, certain genes in the Mehras mitogenome were found to be lacking start codons, as observed in accessions MG372116–MG372121 (NCBI 2023). This discovery supports recent findings in other plant species, where functionally translated mRNAs were found to be devoid of conventional start or stop codons (Planchard et al. 2018). It appears that during plant evolution, unique translational mechanisms have emerged to express mitochondrial genes without relying on the traditional start or stop codons. These distinctive structural features may involve specific ribosome-supporting trans-factors that are dedicated to the translation of plant mitogenomes (Kwasniak-Owczarek et al. 2019).

**Figure 3.**
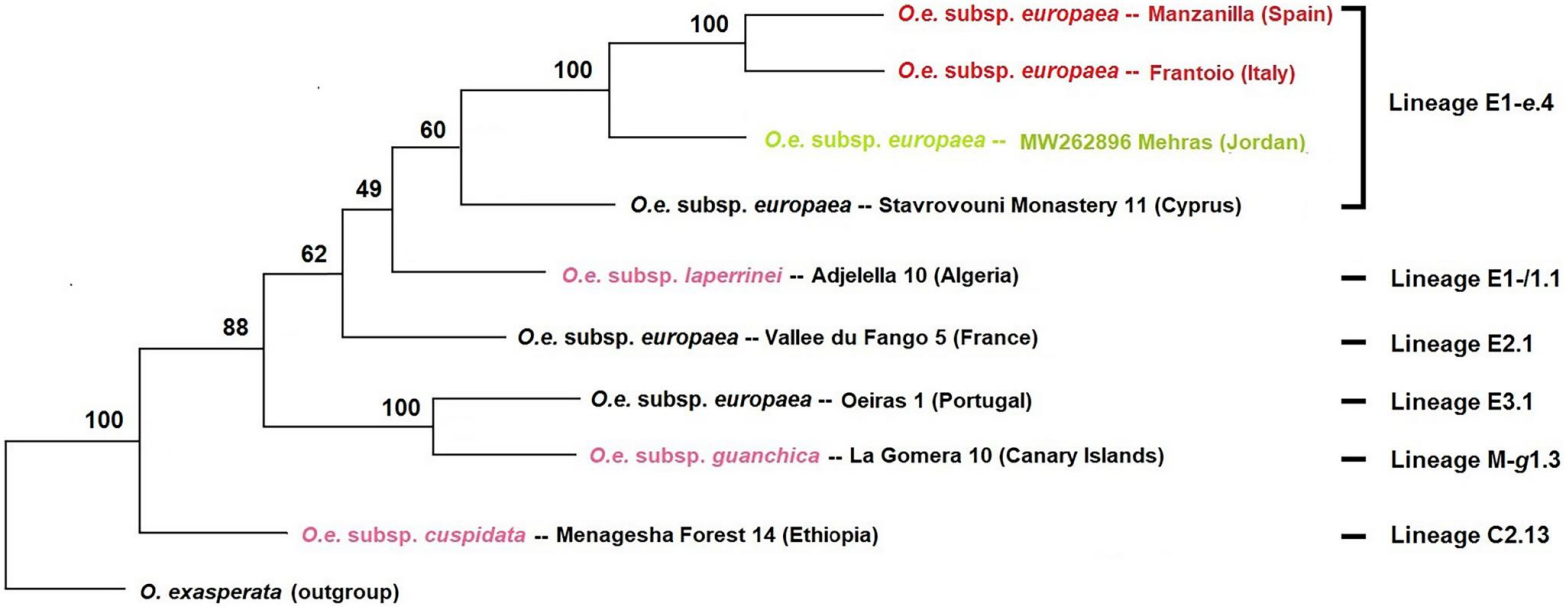
Maximum-likelihood phylogenetic tree of Mehras along with other cultivars (Manzanilla (SRX6614320), Frantoio (SRX6614303), Stavrovouni Monastery 11 (MG372117), Vallee du Frango (MG372118), Oeiras (MG372119)), and related species (*O. e.* L. subsp. *laperrinei* (MG372121), *O. e.* L. subsp. *guanchica*, (MG372120), and *O. e.* L. subsp. *cuspidata* (MG372116)) based on 52 mitochondrial protein-coding genes and introns. Percentage bootstrap values are given on each branch (1000 runs).

The mitogenome of Mehras from Jordan was compared to Stavrovouni Monastery 11 from Cyprus to identify single nucleotide polymorphisms (SNPs) and insertions/deletions (InDels) (see Supplementary Table 1). A total of 925 SNPs were detected, with 564 located in intergenic regions and 361 within intragenic regions. Notably, SNPs were observed in various tRNA genes, such as trnF-GAA (refer to Supplementary Figure 3). Furthermore, additional SNPs were scattered throughout other genes, including ATP subunit genes. In terms of InDels, a total of 68 were detected, primarily distributed across intergenic regions (see Supplementary Table 1).

The phylogenetic tree (Figure 2) revealed that Mehras shares a common ancestor with the Frantoio (Italy) and Manzanilla (Spain) cultivars, which supports earlier findings based on plastome sequencing (Haddad et al. 2021). This finding aligns with Mehras’ historical origin. However, based on plastome data, Mehras (Jordan) exhibits an earlier evolutionary divergence compared to Stavrovouni Monastery 11 (Cyprus) (Haddad et al. 2021). Conversely, the mitogenome analysis (Figure 2) demonstrates a different pattern. Nonetheless, Mehras and Stavrovouni Monastery 11 still share an ancient common ancestor, with a robust bootstrap value of 100% based on plastome data, while the bootstrap value is moderately lower at 60% based on mitogenome data. It is worth noting that the plastome-based results are more reliable as they encompass the entire sequence, including both intergenic and intragenic regions, whereas the mitogenome-based results only cover a partial mitogenome sequence (intragenic regions).

Like Frantoio, Mehras is a dual-purpose cultivar with medium fruit weight (ovoid shape) and medium vigour tree. On the other hand, Manzanilla is a dual-purpose cultivar with high fruit weight (spherical shape) and low vigour tree. However, Mehras fruit oil content is higher than those of either Frantoio or Manzanilla (IOC 2000; Ayoub 2020).

Only three complete mitogenomes for *O. e.* L. subsp. *europaea* were available in the GenBank (NCBI 2023): Oeiras 1 (Portugal), Vallee du Fango 5 (France), and Stavrovouni Monastery 11 (Cyprus). To compare gene order and reading directions, a linear comparison was conducted (see Supplementary Figure 4). The analysis revealed complete synteny between Stavrovouni Monastery 11 and Mehras, whereas apparent differences were observed for Oeiras 1 and Vallee du Fango 5. These findings confirmed the established lineages E1–e4 (Stavrovouni Monastery 11 and Mehras), E3.1 (Oeiras 1), and E2.1 (Vallee du Fango 5) (Van de Paer et al. 2018).

## Supplementary Material

Supplemental Material

Supplemental Material

Supplemental Material

Supplemental Material

Supplemental Material

## Data Availability

The genome sequence data that support the findings of this study are openly available in GenBank of NCBI at https://www.ncbi.nlm.nih.gov under the accession number MW262896. The associated BioProject, Bio-Sample, and SRA numbers are PRJNA670825, SAMN16521220, and SRX9347605–SRX9347608, respectively.
