# Supplementary figures and images for: Complete mitochondrial genome sequence of historical olive (Olea europaea Linnaeus 1753 subsp. europaea) cultivar Mehras in Jordan
Source: Mitochondrial DNA B Resour. 2023 Nov 7;8(11):1205–8. doi: 10.1080/23802359.2023.2275828 (PMC10796119; doi:10.1080/23802359.2023.2275828)

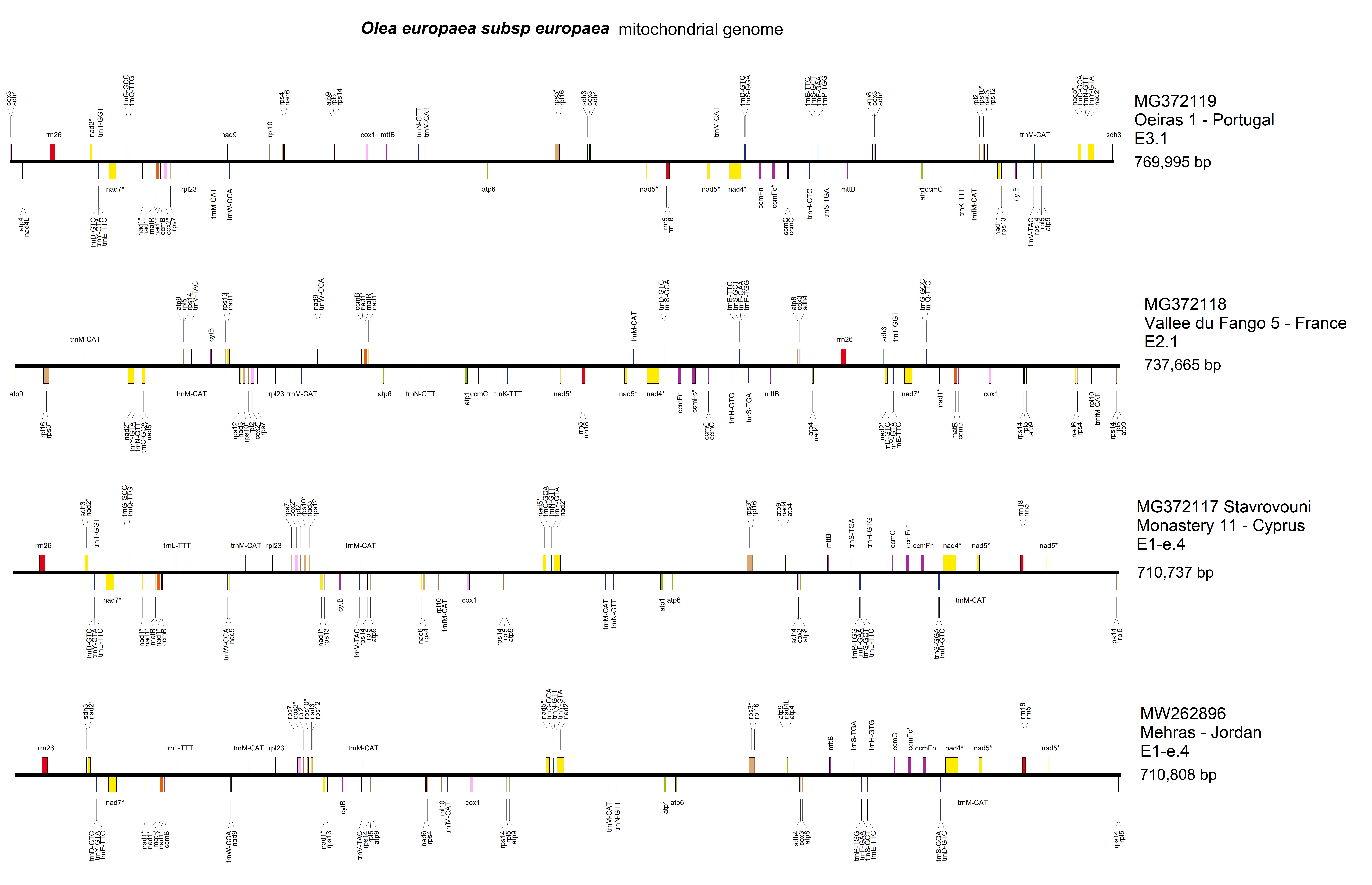

Supplement: Supplemental Material [file TMDN_A_2275828_SM6104.jpg]

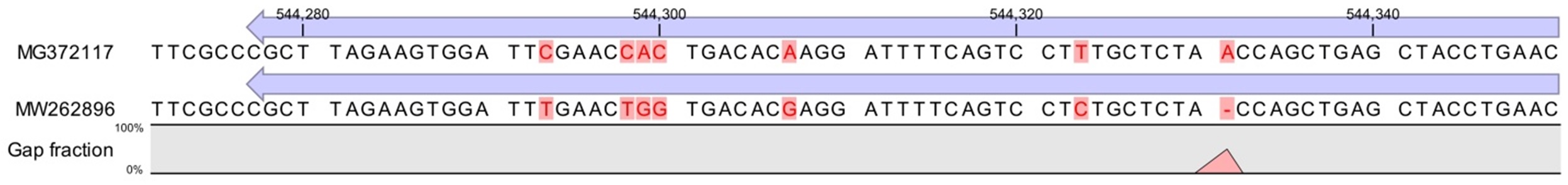

Supplement: Supplemental Material [file TMDN_A_2275828_SM6103.jpg]

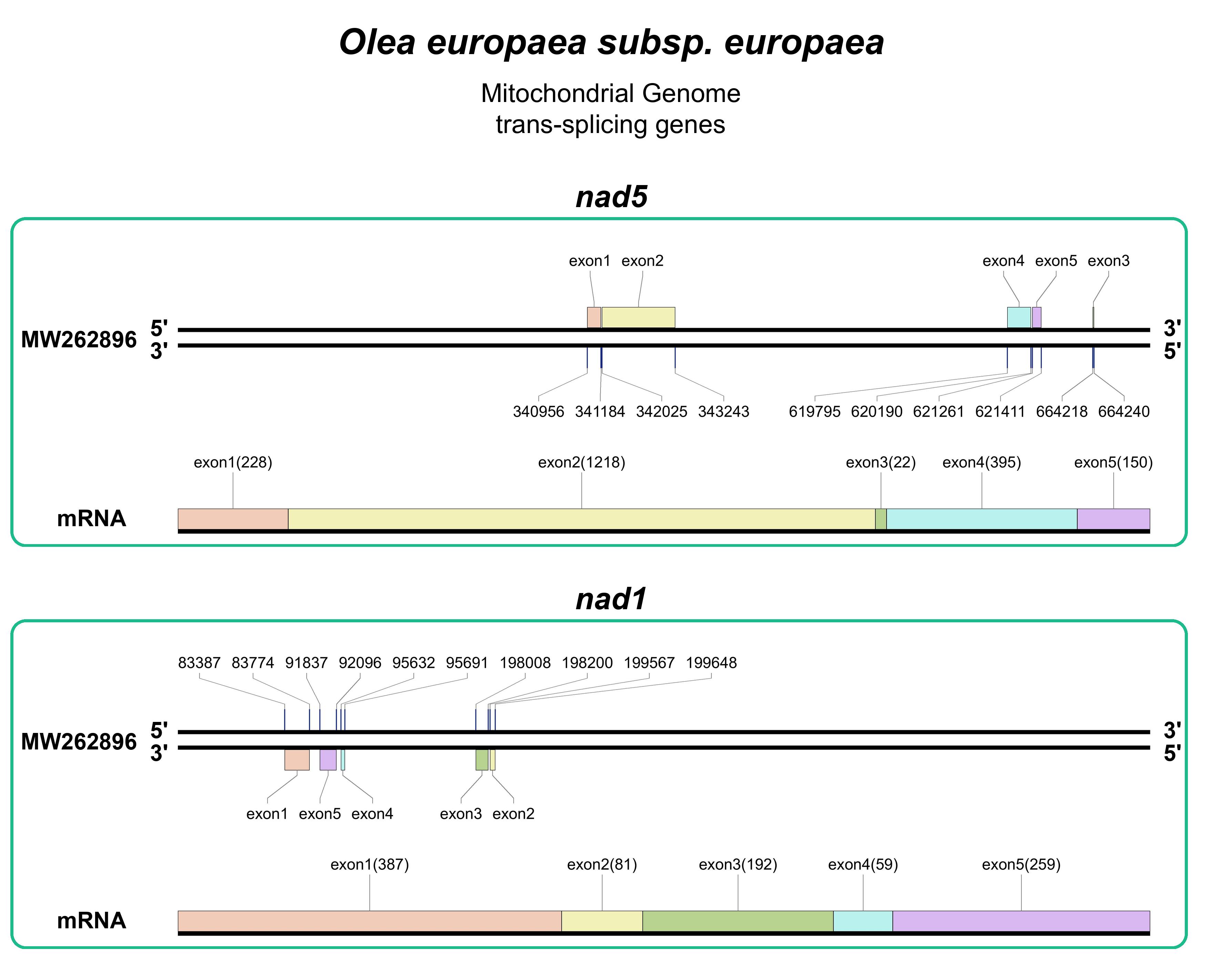

Supplement: Supplemental Material [file TMDN_A_2275828_SM6102.jpg]

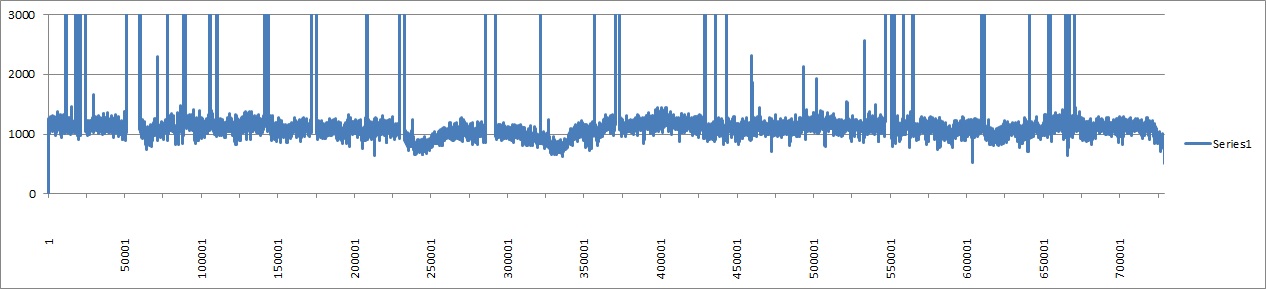

Supplement: Supplemental Material [file TMDN_A_2275828_SM6091.jpg]
