# Supplementary material for: Complete mitochondrial genome sequence of historical olive (Olea europaea Linnaeus 1753 subsp. europaea) cultivar Mehras in Jordan
Source: Mitochondrial DNA B Resour. 2023 Nov 7;8(11):1205–8. doi: 10.1080/23802359.2023.2275828 (PMC10796119; doi:10.1080/23802359.2023.2275828)
Supplement: Supplemental Material [file TMDN_A_2275828_SM6089.docx]

**Complete mitochondrial genome sequence of historical olive (*Olea europaea* L. subsp. *europaea*) cultivar Mehras in Jordan**

Monther Sadder ^a*^, Mohammad Brake ^b^, Salam Ayoub ^c^, Yahya Abusini ^c^, Ibrahim Al-Amad ^c^, Nizar Haddad ^c^

^a^ School of Agriculture, The University of Jordan, Amman, Jordan

^b^ Science Faculty, Jerash University, Jordan

^c^ National Agricultural Research Center - NARC, Baqa'a, Jordan

* Correspondence:

Monther Sadder

[sadderm@ju.edu.jo](mailto:sadderm@ju.edu.jo)

**Supplementary** **Table 1.** Available mitogenome SNPs and InDels detected between *O.e.* L. subsp. *europaea* cultivar Mehras (Jordan) and related cultivar Stavrovouni Monastery 11 (Cyprus).

| **Region** | **Gene name** | **Gene product** | **SNPs** | **InDels (bases)** | | | | | | | |
| --- | --- | --- | --- | --- | --- | --- | --- | --- | --- | --- | --- |
|  |  |  |  | **1** | **2** | **3** | **4** | **5** | **6** | **7** | **8** |
| Intergenic |  |  | 564 | 23 | 10 | 5 | 7 | 5 | 5 | 3 | 1 |
| Intragenic | rrn26 | 26S ribosomal RNA | 189 | 2 | 1 |  | 1 |  |  |  |  |
|  | rrn18 | 18S ribosomal RNA | 120 | 1 |  |  |  |  |  |  |  |
|  | trnW-CCA | tRNA-Trp | 1 |  |  |  |  |  |  |  |  |
|  | rpl23 | ribosomal protein L23 | 2 |  |  |  |  |  |  |  |  |
|  | trnV-TAC | tRNA-Val | 4 | 1 |  |  |  |  |  |  |  |
|  | trnM-CAT | tRNA-Met | 1 |  |  |  |  |  |  |  |  |
|  | trnN-GTT | tRNA-Asn | 3 |  |  |  |  |  |  |  |  |
|  | atp1 | ATPase subunit 1 | 15 |  |  |  |  |  |  |  |  |
|  | atp4 | ATPase subunit 4 | 1 |  |  |  |  |  |  |  |  |
|  | trnS-TGA | tRNA-Ser | 13 |  |  | 2 |  |  |  |  |  |
|  | trnF-GAA | tRNA-Phe | 5 | 1 |  |  |  |  |  |  |  |
|  | trnH-GTG | tRNA-His | 3 |  |  |  |  |  |  |  |  |
|  | trnS-GGA | tRNA-Ser | 3 |  |  |  |  |  |  |  |  |
|  | trnD-GTC | tRNA-Asp | 1 |  |  |  |  |  |  |  |  |
| Total |  |  | 925 |  |  |  |  |  |  |  | 68 |

**
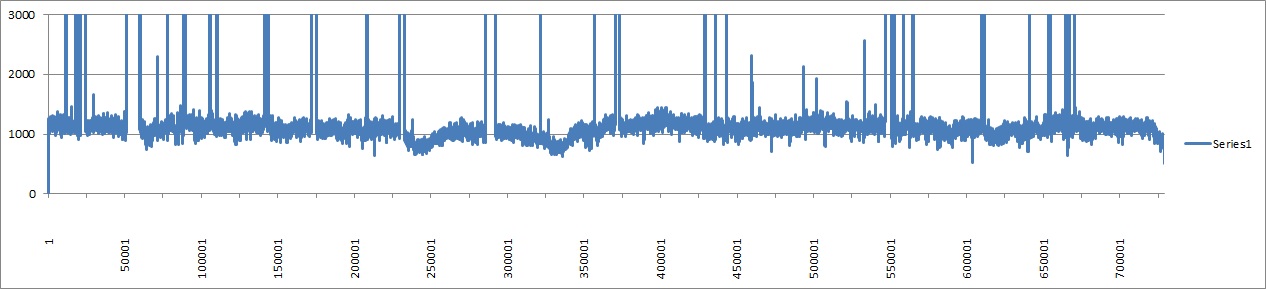
**

**Supplementary** **Figure 1.** Sequencing depth and coverage map.

**
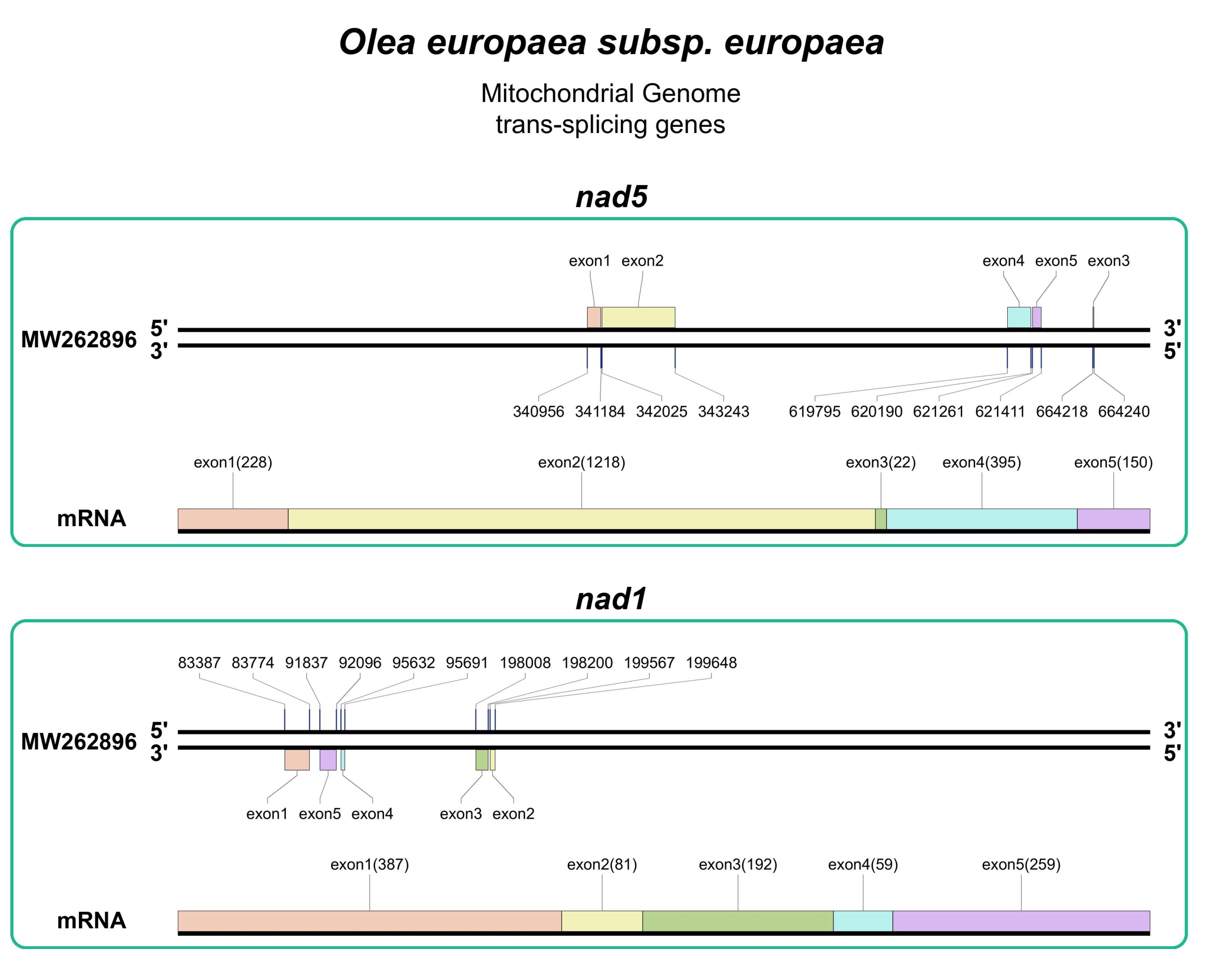
**

**Supplementary** **Figure 2.** Map showing the structure of complex genes.

**
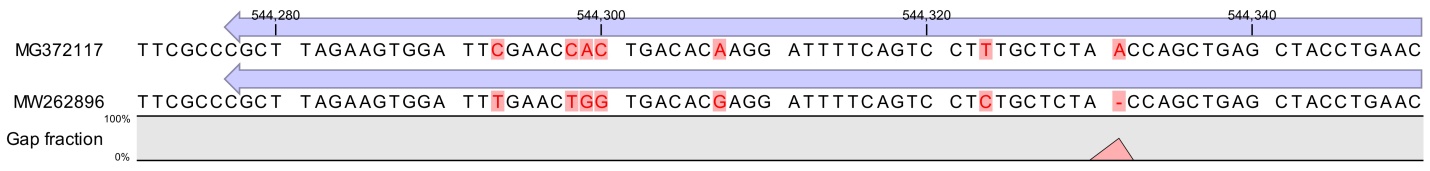
**

**Supplementary** **Figure 3.** Alignment between *O.e.* L. subsp. *europaea* cultivar Mehras (Jordan, MW262896) and related cultivar Stavrovouni Monastery 11 (Cyprus MG372117) for mitochondrial gene trnF-GAA showing three SNPs and one InDel.

**
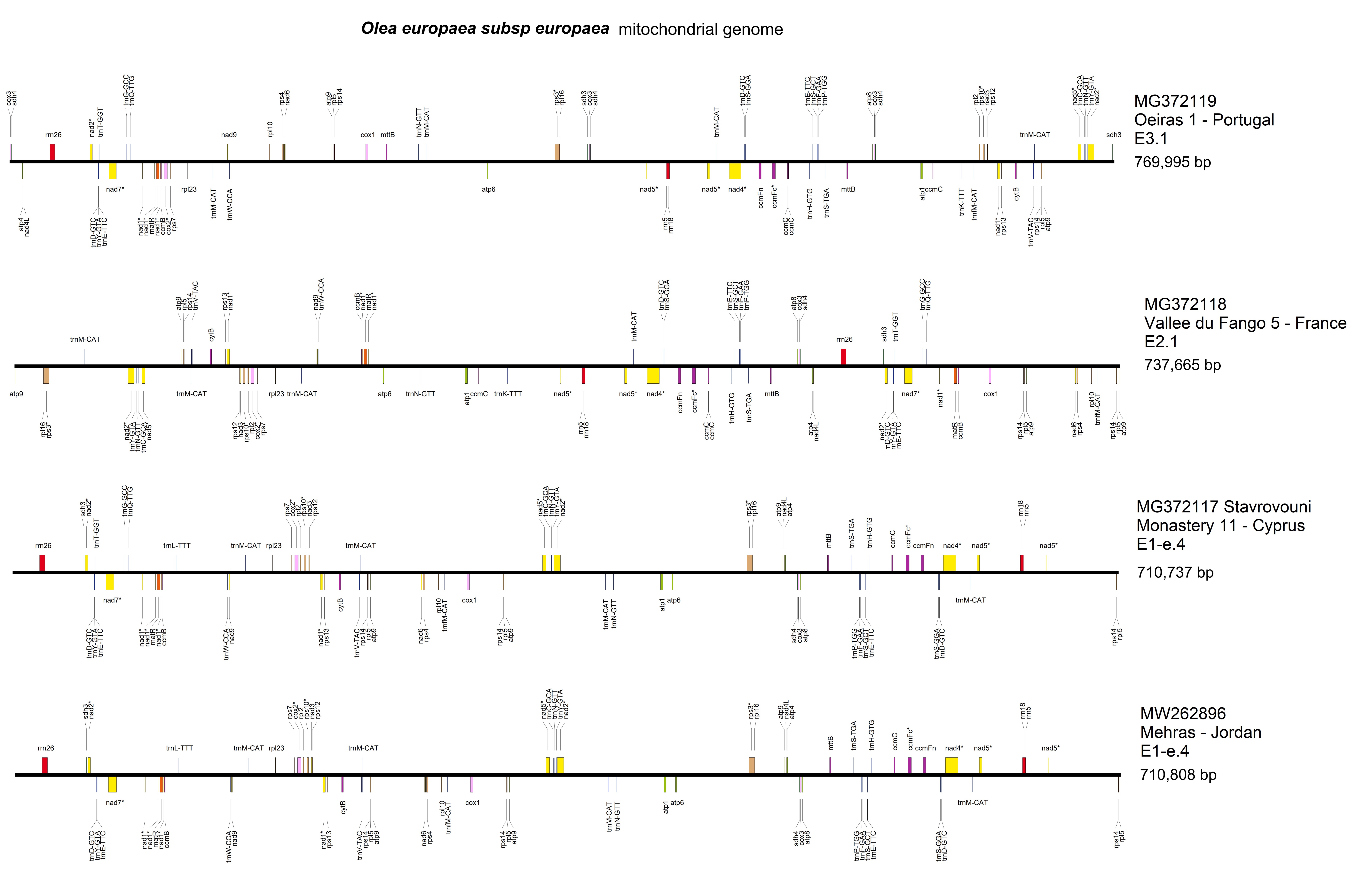
**

**Supplementary** **Figure 4.** Comparison of mitochondrial maps showing gene order and reading direction between four cultivars of *O.e.* L. subsp. *europaea*.
